# Development of an Enhanced Recovery Program in Pediatric, Adolescent, and Young Adult Surgical Oncology Patients

**DOI:** 10.3390/children8121154

**Published:** 2021-12-08

**Authors:** Stephanie J. Wells, Mary Austin, Vijaya Gottumukkala, Brittany Kruse, Lauren Mayon, Ravish Kapoor, Valerae Lewis, Donna Kelly, Alexander Penny, Brent Braveman, Eliana Shkedy, Rebekah Crowder, Karen Moody, Maria C. Swartz

**Affiliations:** 1 Department of Pediatrics Research, Division of Pediatrics, The University of Texas MD Anderson Cancer Center, Houston, TX 77030, USA; sjwells1@mdanderson.org; 2 Department of Surgical Oncology, The University of Texas MD Anderson Cancer Center, Houston, TX 77030, USA; maustin@mdanderson.org (M.A.); lkmayon@mdanderson.org (L.M.); 3 Department of Anesthesiology and Perioperative Medicine, The University of Texas MD Anderson Cancer Center, Houston, TX 77030, USA; vgottumukkala@mdanderson.org (V.G.); rkapoor@mdanderson.org (R.K.); 4 Institute for Cancer Care Innovation, The University of Texas MD Anderson Cancer Center, Houston, TX 77030, USA; bccampbell@mdanderson.org; 5 Department of Orthopaedic Oncology, The University of Texas MD Anderson Cancer Center, Houston, TX 77030, USA; volewis@mdanderson.org (V.L.); anpenny@mdanderson.org (A.P.); 6 Department of Rehabilitation Services, The University of Texas MD Anderson Cancer Center, Houston, TX 77030, USA; DMKelly@mdanderson.org (D.K.); bhbraveman@mdanderson.org (B.B.); 7 Department of Clinical Nutrition, The University of Texas MD Anderson Cancer Center, Houston, TX 77030, USA; ebshkedy@mdanderson.org (E.S.); racrowder@mdanderson.org (R.C.); 8 Department of Patient Care, Division of Pediatrics, The University of Texas MD Anderson Cancer Center, Houston, TX 77030, USA; kmoody@mdanderson.org

**Keywords:** enhanced recovery, pediatrics, cancer, surgical oncology, pain management

## Abstract

Enhanced recovery after surgery (ERAS) protocols are standardized perioperative treatment plans aimed at improving recovery time in patients following surgery using a multidisciplinary team approach. These protocols have been shown to optimize pain control, improve mobility, and decrease postoperative ileus and other surgical complications, thereby leading to a reduction in length of stay and readmission rates. To date, no ERAS-based protocols have been developed specifically for pediatric patients undergoing oncologic surgery. Our objective is to describe the development of a novel protocol for pediatric, adolescent, and young adult surgical oncology patients. Our protocol includes the following components: preoperative counseling, optimization of nutrition status, minimization of opioids, meticulous titration of fluids, and early mobilization. We describe the planning and implementation challenges and the successes of our protocol. The effectiveness of our program in improving perioperative outcomes in this surgical population could lead to the adaptation of such protocols for similar populations at other centers and would lend support to the use of ERAS in the pediatric population overall.

## 1. Introduction

Enhanced recovery after surgery (ERAS) programs aim to use evidence-based practices to improve perioperative care. Using a multidisciplinary, patient-focused approach, ERAS programs strive to minimize the physiologic stress associated with selected surgeries in certain patient populations. There is abundant literature describing successful implementation of ERAS programs in adults. Although growing, the evidence in pediatric surgical populations is not as robust [1].

Patients undergoing oncologic surgery may be particularly at risk of perioperative complications due to their pathology, as well as the side effects of cancer treatment. In fact, surgery for oncologic indications is particularly associated with increased surgical complications [2]. To our knowledge, no ERAS-based protocols have been described in the literature specifically for pediatric cancer patients, despite the persistence of surgical complications in this vulnerable population [3]. We aim to describe the development of an enhanced recovery program for children, adolescents, and young adults undergoing major abdominal, thoracic, or orthopedic oncologic surgery at our comprehensive cancer care institution.

## 2. Materials and Methods

We adapted the ERAS protocol that fits the workflow at MD Anderson Cancer Center (MDACC). Henceforth, we will refer to the ERAS-based protocols at MDACC as enhanced recovery program (ERP) protocols. Key stakeholders were assembled to discuss a plan for the ERP including pediatric surgeons, anesthesiologists, quality improvement specialists, pediatric pain management/supportive care physicians, nurse practitioners, clinical dietitians, research dietitians, behavioral researchers, physical therapists, occupational therapists, psychologists, and patient education experts. Once consensus was reached on the program components, adult ERP protocols at MDACC were modified to meet the needs of the younger surgical population. For this protocol, we focused on key elements of the ERP pathway that we needed to improve (Table 1) since many of the components of the original ERAS^®^ guidelines [4], such as antibiotic and thromboprophylaxis, were already normalized in our practice. Where information was lacking for this age group, we based our guidelines on published evidence from other institutions, expert opinions, and clinical relevance. We included young adult patients followed in the Pediatric Intensive Care Unit after surgery. Protocol-specific order sets were integrated into the electronic medical record in order to streamline the process for ordering analgesics. We modified our surgical order sets to remove orders for opioids and to include consultations on physical and occupational therapy, nutrition, child life, psychology, and supportive care/pain management. We also created a new order set for postoperative pain medications, to be utilized by the supportive care and acute pain teams. Patient education materials were created to address expectations regarding mobility, pain, consumption of carbohydrate-containing clear beverages prior to surgery, and other program components. Education of anesthesia staff, post-anesthesia recovery unit nurses, pediatric intensive care unit staff, and the pain management team was achieved during routine staff meetings. A banner in our EMR was created to identify a patient on the ERP protocol. The IRB at MDACC approved our written protocol for extracting the data and reporting on our ERP experience. A “soft launch” was conducted, using the first three surgical cases to test the logistics of the protocol procedures.

## 3. Results

The timing of our ERP protocol for pediatric, adolescent, and young adult surgical oncology patients is outlined in Table 1 and described herein. Before surgery, caregivers and patients are educated by an advanced practice provider on ERP principles, patient/caregiver expectations, and pain management, in addition to viewing a video on general perioperative information. The purpose of this additional education is to improve adherence to the ERP protocol, address any concerns on the part of the patient and/or caregivers, and ensure that the patient and caregivers are fully aware of what is expected of them.

Prior to surgery, patients are referred to pain management/supportive care and child life. They are also referred to clinical psychology if surgery is expected to result in changes to body appearance or function or if requested by the patient or family. Patients are screened based on cancer type and body weight for height to determine malnutrition risk. Screening is performed using the patient needs screening tool, which contains a modified malnutrition screening tool developed at MDACC. This tool includes four questions: (1) decreased appetite in the past two weeks, (2) weight loss > 10 pounds in the past month, (3) receiving tube feeding or total parenteral nutrition, and (4) patient has a diet- or nutrition-related question for the clinical dietitians. When at least one of these questions is answered affirmatively, a nutrition assessment is conducted by a clinical dietitian and interventions to optimize the patient’s nutrition status prior to surgery are recommended. On the day before surgery, consumption of fluids and a well-balanced dinner including protein-rich foods is advised. Patients are also directed to consume a carbohydrate-containing clear beverage, such as a sports drink or fruit juice, on the night before surgery and again 2 h before surgery, for a total of two doses. The recommended volume is 3–5 mL per kg of body weight [5,6] per dose. On the day of surgery, patients are allowed to drink clear liquids ad libitum until 2 h prior to surgery. At that time, they are advised to drink their second carbohydrate-containing clear beverage dose. Patient education handouts can be found in the Supplementary Materials (Files S1–S5).

Analgesics are provided perioperatively with doses adjusted by age, renal function, and liver function. No medications are used preoperatively. Patients undergoing major abdominal/thoracic surgery are not given preoperative analgesics, as similar medications can be administered intravenously with more appropriate timing intraoperatively. An opioid-sparing strategy using multimodal analgesia is used during surgery. These analgesics typically include intravenously administered acetaminophen, ketorolac, and ketamine/dexmedetomidine infusions as appropriate. Regional blocks in the form of thoracic epidurals or local wound infiltration with long-acting liposomal bupivacaine are used in selected patients undergoing major abdominal/thoracic surgery, to minimize opioid administration. Regional/neuraxial analgesia and long-acting liposomal bupivacaine blocks are generally avoided in orthopedic surgery patients at our center due to the need for close monitoring of hemodynamic instability as well as sensory and motor function. Postoperatively, a protocol-based, risk-stratified, multimodal approach is followed. Acetaminophen is used routinely on all patients, ketorolac is used when not contraindicated, neuropathic agents and muscle relaxers are employed when appropriate, and interval opioids are used for breakthrough pain. In patients without regional blocks who are expected to have severe pain (e.g., hemipelvectomy), low-dose methadone is also utilized. Use of postoperative drains such as nasogastric tubes and urinary catheters are avoided if possible. Foley catheters, previously routinely left in place to prevent urinary retention during epidural use, are now removed at the end of the surgery.

Following surgery, patients are encouraged to move out of bed to a chair on postoperative day 0 and ambulate the day after surgery. They are followed closely by the nutrition, occupational therapy, physical therapy, supportive care/pain management, and child life teams. Physical and occupational therapy teams are consulted on the day of surgery, and they assist with early and safe mobility. Movement out of bed to a chair is encouraged, with ambulation at least six times daily. After surgery, early oral nutrition is the goal. Dietitians recommend clear or full liquids at first, with rapid progression to a regular diet. Oral nutrition supplements such as meal replacement shakes are recommended as appropriate. For thoracic surgical patients, a regular diet is started immediately after surgery. For patients undergoing bowel surgery, clear liquids are given on the day of surgery, followed by an advancement of diet as tolerated. The acute pain service co-manages the patients’ postoperative analgesia needs when continuous regional blocks are used perioperatively. Once blocks are removed, or if there are no blocks, the pediatric supportive care service provides pain management for the remainder of the admission period. Discharge criteria vary based on the type of surgery, but generally include pain well controlled with oral medications, tolerating a regular diet, ability to ambulate, and removal of drains. Pain management orders are listed in Table 2.

During the “soft launch”, there were four pediatric ERP surgeries in three patients: Patient #1 with a left chest wall resection, Patient #2 with staged thoracotomies, and Patient #3 with a left thoracotomy, all of whom experienced positive outcomes. Patient #1 did not require any opioids, and the other two patients required two or fewer doses of opioid during the entire length of stay. Additionally, Patients #1 and #3 went home on postoperative day two. This is earlier than in previous studies, where lengths of stay (LOS) ranging from 4.3 to 5 days were reported for thoracotomies in pediatric, adolescent, and young adult patients [7,8,9]. The staged thoracotomy patient (Patient #2) was discharged on postoperative day three and day five, respectively, after each thoracotomy. This patient had 29 wedge resections and had an air leak postoperatively following the second thoracotomy, which increased the patient’s LOS. However, his LOS was average compared to our pre-ERP outcomes.

Several challenges were noted during the “soft launch” period. Although education for patient care providers (e.g., nurses, oncologists, pharmacists) related to the pediatric ERP procedures was completed prior to the “soft launch,” we identified opportunities for improvement. These opportunities were in the areas of staff education, family education, and tracking adherence to ERP procedures. These challenges and their solutions are described in more detail in Table 3.

## 4. Discussion

To the best of our knowledge, this is the first enhanced recovery protocol created for pediatric, adolescent, and young adult surgical oncology patients. Despite the scarcity of pediatric ERAS protocols, there is evidence to suggest they are safe and may be effective in decreasing LOS and opioid use without increasing surgical complications [3,4]. While many more ERP cases are needed in order to determine the effectiveness of our program, our preliminary results seem to support these findings, as two ERP cases were discharged home on postoperative day two, and no or minimal opioids were required for pain management. While evidence for the effectiveness of ERAS protocols in pediatric cancer patients is lacking, positive results seen in other pediatric surgeries are encouraging. A recent ERAS protocol in pediatric GI surgery patients resulted in significant improvements in bowel function recovery time, postoperative parenteral nutrition time, postoperative LOS, and hospital costs, with no significant difference in infection rate between the ERAS and control groups [5]. Similarly, another study found significantly higher rates of same-day discharge and reduced opioid use, with no increase in emergency room use or readmissions after ERAS implementation [6]. These results suggest that post-surgical recovery and quality of care can be improved when adhering to a pediatric ERAS protocol. It is expected that many of these benefits will translate to pediatric cancer patients as well.

The lack of ERAS-based protocols for pediatric cancer patients is an opportunity for improvement, considering the presence of surgical complications in this population. Gallaway et al. found that 1 in 11 pediatric bone and soft tissue sarcoma surgical patients experienced complications within 30 days post-surgery, including wound dehiscence, surgical site infections, pneumonia, urinary tract infections, C. diff colitis, and unplanned readmissions related to the surgical procedure [10]. Additionally, 24% of patients in this study required blood transfusions due to excessive bleeding. These findings highlight the importance of developing ERAS protocols tailored to the pediatric surgical oncology population in order to minimize such complications.

There are numerous reasons why ERAS protocols are effective. One reason may be their focus on addressing multiple variables affecting anabolic homeostasis. Malnutrition, including extremes of both undernutrition and overnutrition, is one of these essential perioperative considerations. The prevalence of malnutrition varies based on cancer type [11], ranging from 10 to 50% in pediatric cancer patients [12], and may be as high as 75% in adolescent and young adult (AYA) cancer patients [13]. Malnutrition has been shown to negatively impact phase I and II clinical trial outcomes in adult cancer patients [14] and chemotherapy tolerance [15] and survival rate in pediatric cancer patients [16]. These negative effects of malnutrition highlight the importance of assessing and intervening in nutrition status before, during, and after surgery, by optimizing early enteral nutrition. Other important nutritional components include carbohydrate loading prior to surgery and avoidance of perioperative fasting, which have been shown to significantly reduce postoperative nausea [6], loss of lean body mass, and surgically induced insulin resistance [17]. Another consideration for optimizing anabolic homeostasis is early mobilization. Ambulation has been shown to shorten recovery time and maintain physical function [18], prevent muscle atrophy and deconditioning [19], and possibly decrease postoperative morbidity [20]. Optimizing and standardizing pain management protocols plays a critical role in early mobility [21].

Going forward, the success of our ERP protocol is likely to require more iterations of the procedures and processes, consistent coordination of care, effective communication among clinical staff, and re-training on protocol procedures. This is supported by findings from a study examining an ERAS protocol for colorectal resection, in which most protocol deviations occurred postoperatively and discharge did not occur until 2 days following functional recovery [22]. It will also be important to closely monitor the recovery of each patient to prevent readmissions resulting from premature discharge home [22], using an ERP-based dashboard to track the major outcomes (e.g., pain scores, opioid type and use, LOS, quality of life, comorbid complications, etc.). If effective in improving perioperative outcomes in pediatric, adolescent, and young adult surgical oncology patients, our ERP may be adapted for other pediatric cancer patient populations such as pediatric hematopoietic stem cell transplant recipients.

## Figures and Tables

**Table 1:** ERP components.

Time Point	ERP Components
Preoperative	Education for caregivers and patients on ERP components and expectations Nutrition screening to determine malnutrition risk and assessment by registered dietitians when needed to provide appropriate interventions Nutrition education provided on preoperative carbohydrate-containing clear beverage consumption (avoidance of prolonged fasting) by advanced care practitioners and registered dietitians
Intraoperative	Opioid minimization Multimodal analgesia Incorporation of regional anesthesia when possible Maintenance of normothermia Goal-directed fluid therapy
Postoperative	Mimization of postoperative drains Early mobilization Early use of physical/occupational therapy Early oral nutrition/diet progression Scheduled multimodal analgesics

**Table 2:** ERP pain management orders.

Order Set	Non-Opioid Medications	Opioids Medications
Low- and medium-dose opioid set *	Acetaminophen 12.5–15 mg/kg/dose IVPB q 6 h	Oral or IV PRN for moderate or severe pain ^‡^: Hydromorphone Morphine Oxycodone
	Ketorolac IV (for patients ≥ 2 years old) and no contraindication 0.5 mg/kg q 6 h (max 15 mg)	
	Regional/neuraxial blockade	
	Methocarbamol IV 10 mg/kg q 8 h as needed	
High-dose opioid set ^†^	Acetaminophen 12.5–15 mg/kg/dose 0.5 mg/kg IVPB q 6 h	Oral or IV PRN for moderate or severe pain: Methadone Hydromorphone Morphine Oxycodone
	Ketorolac IV (for patients ≥ 2 years old) and no contraindication	
	Methadone 0.05 mg/kg po q12 h (max 2.5 mg q 12)	
	Methocarbamol IV 10 mg/kg q 8 as needed	
Transition to oral dosing	Methocarbamol po (for patients > 4 years old)	Oral medications PRN for moderate or severe pain: Hydromorphone Morphine Oxycodone
	Baclofen oral suspension if unable to swallow methocarbamol pills	
	Gabapentin 5 mg/kg po q 12	

* Neuraxial/regional analgesia in place and/or patients undergoing craniotomy, ophthalmology procedures, thoracotomy, etc. ^†^ For patients such as orthopedic surgery patients, including hemipelvectomy, or patients without neuroaxial/regional analgesia. ^‡^ Pain is considered moderate with a pain score of 4–6 and is considered severe with a pain score of 7–10.

**Table 3:** Challenges faced during ERP testing.

Challenge		Solution
Staff education	Training initially provided to nursing staff was inadequate as evidenced by confusion over which preoperative medications to provide	Provide more detailed staff training prior to formal launch
		Conduct ongoing staff education throughout implementation of the ERP
Family education	Inconsistencies between education provided verbally to families and education provided in handouts	Education handouts were revised prior to the formal ERP launch
	First preoperative education session was done virtually where the educator only spoke directly with the parent. This required sending the educational handouts through the electronic medical record portal	Conducting education sessions in person or scheduling a video call only when the caregiver and patient will both be present
Tracking adherence to ERP procedures	The process for alerting nursing staff, oncology team, pharmacy, and consulting services about scheduled ERP patients was disorganized	Create an email group of ERP champions in each clinical area and send out an email to alert them prior to each ERP patient surgery
	Adherence to ERP procedures was difficult to determine as there was no tracking system for this purpose	Create an ERP-specific dashboard in the electronic medical record that tracks adherence outcomes

## Supplementary Materials

The following are available online at https://www.mdpi.com/article/10.3390/children8121154/s1, File S1: Enhanced recovery after surgery: pediatric pain management information and resources; File S2: Enhanced recovery after surgery: pediatric patient; File S3: Enhanced recovery program overview for pediatric patients; File S4: Nutrition—pediatric enhanced recovery after surgery; File S5: Prehabilitation—pediatric enhanced recovery after surgery.
