# Supplementary material for: Development of an Enhanced Recovery Program in Pediatric, Adolescent, and Young Adult Surgical Oncology Patients
Source: Children (Basel). 2021 Dec 8;8(12):1154. doi: 10.3390/children8121154 (PMC8700533; doi:10.3390/children8121154)
Supplement: Supplementary file 1 [file children-08-01154-s001.zip › File S1.pdf]

Enhanced Recovery After Surgery

Pediatric Pain Management Information and Resources

Overview

This handout provides information to help you and your **child, adolescent** or **young adult (CAYA)** prepare for surgery.

It is important that you and your CAYA partner with the medical care team to help prepare for surgery. Members of the care team include surgeons, nurses, pain specialists, child life specialists, physical therapists and sometimes others. The care team will listen and work with both of you to develop a plan for managing pain, which includes keeping pain under control.

The main thing to know is that if your CAYA reports pain, the care team will take action to comfort your CAYA and treat and manage the pain.

Communicating Pain

Only the person with pain knows how much pain they have. They need to be able to describe their pain to the care team. Using a rating scale is helpful to describe how much pain a person feels.

The care team uses a numeric pain rating scale called the **Wong Baker Faces Scale** to assess pain.

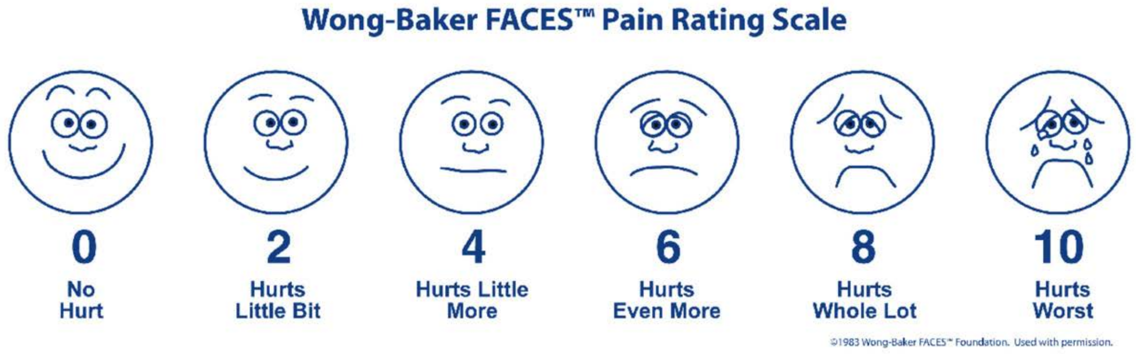

The University of Texas MD Anderson Cancer Center ©2021

Emotional and Mental Distress

Emotions

The mind and body work together. This is especially true when it comes to managing pain. Sometimes, how a person feels (emotions) can increase or decrease pain and may be related to certain symptoms.

Emotional and mental changes are common during treatment for a serious illness and may be a sign of emotional pain or physical illness.

Services and resources are available to help ease or improve emotional pain and distress. Be sure to tell the care team about any of these signs and symptoms:

- Mood changes, such as feeling irritable, low, or sad
- Anxiety
- Behavior changes
- Relationship changes (withdrawn)
- Sleep changes, such as problems falling or staying asleep, or having nightmares
- Appetite changes
- Having bad or upsetting thoughts
- Feeling hopeless or helpless

Coping

Every person has a sense of self and how to help oneself through life challenges. People use certain strategies specific to their life challenges. Illness, medical procedures and treatments may present new challenges. These may require developing new coping strategies. For example, being positive (optimistic) is a strategy that tends to improve mood. It is an outlook or habit where a person focuses on the positive aspects of any situation.

Services and Resources

There are available services and resources, such as social work and psychosocial services, that you and your CAYA can use for help with certain strategies.

Pediatric Supportive Care Team

The Pediatric Supportive Care team specializes in pain and symptom management. The main role of this team is to manage your child's pain after surgery using the most up-to-date medicine management and non-medicine strategies. This team includes doctors, nurse practitioners and a nurse.

Child Psychology Team

The Child Psychology team can provide one-on-one counseling to help ease pain and anxiety related to surgery. CAYAs with pre-existing anxiety who are having a major surgery will receive a referral to the Child Psychology team. Family/caregivers may also request a referral.

Self-Soothing Activities

Self-soothing activities can help people feel calm, stable and enhance inner strength when feeling anxious or scared, or going through illness, treatment and recovery. Activities can include breathing exercises, music, distraction and use of touch and smell, such as aromatherapy and essential oils. Other options include using smartphone apps, such as Calm®.

Spiritual Support

Chaplains through Spiritual Care and Education are available 24 hours a day for spiritual support and serve people of many spiritual backgrounds. If you would like to speak with a chaplain, tell the care team.

Child, Adolescent, and Young Adult Life Specialist

Every child, adolescent and young adult patient having surgery will receive a referral to see a **Child, Adolescent and Young Adult Life Specialist**. They teach proven strategies to help young patients and their families cope with and lessen the impact of cancer, procedures and hospital stays.

These strategies may include providing age and developmentally appropriate support for medical procedures, educating patients and caregivers about disease process and treatment options, and engaging patients and building relationships through play.

Specialists also use proven strategies to help with pain management, based on the patient's personal needs. Strategies may include:

- Creating a coping plan before a procedure to help reduce pain after the procedure
- Using play, distraction, breathing exercises, guided imagery and other methods to help lessen or relieve pain

Pediatric Integrative Medicine

Pediatric integrative medicine services for CAYAs focus on the whole person through recovery by engaging the mind, body and spirit. Many types of strategies may be used along with standard medicines to help manage pain, symptoms, and improve quality of life. These strategies are safe, and many have proven to be effective. An integrative medicine consult is available upon request and is also available to parents, siblings and caregivers. Some of the services include:

- **Aromatherapy** involves the use of scents to help with relaxation and elevate mood.
- **Acupuncture** uses tiny needles or magnets to decrease pain and swelling after surgery, help with nausea, promote sleep and decrease the length of hospital stays.
- **Massage therapy** eases muscle tension, provides relaxation, and promotes sleep.
- **Reiki** is a type of energy medicine used to balance the body's energy.
- **Mind-Body** exercises help with the mind and body connection, improving one's effect on the other. When the mind is stressed, the body is often tense. When the mind is at ease, the body tends to relax. Focused attention can help the mind relax, which in turn can help ease body tension and decrease pain. For example, by focusing on a soother, such as a special object or place, positive feelings may be created, and muscles may relax.
- **Lifestyle strategies**, such as nutrition, exercise, and sleep are important to healing after surgery. Your CAYA will receive a referral for nutrition and physical therapy

services. Your CAYA will also receive specific diet instructions 24 hours before surgery.

Sleep

Sleep hygiene strategies can include the following:

- Turn off electronics 30 minutes before bed
- At bedtime, picture a relaxing place that brings to mind sounds, smells and feelings. Apps and online video sites, such as YouTube®, are options if you prefer narrated relaxation exercises.
- Keep a regular bedtime routine, such as a bath and a story.

The Pediatric Integrative Medicine, Child Life, and Child Psychology teams offer more mind-body and sleep management strategies. A mind-body therapist can work one-on-one with your CAYA using meditation techniques, reflexology, and yoga.

Arts in Medicine

The Arts in Medicine team members offer expressive arts activities and music therapy to provide comfort, relaxation, joy, inspiration, social connection, and self-expression. Creative arts can uplift the whole person's body, mind, and spirit. This service is available upon request.

Visual arts activities include group classes, as well as bedside services for CAYAs and families. Self-guided art activities, such as origami, coloring and sticker books, art journal, and virtual reality experiences are also available.

Music Therapy Tips Before Surgery

- Create a playlist of favorite songs on an electronic device (smartphone, mp3 player, or tablet) that helps calm or distract.
- Choose a song to practice deep breathing and use it to encourage breathing before surgery.
- Engage in active music making with instruments from home or use apps on a device. This can help distract, provide comfort and encourage family-child bonding.
- Ask for a music therapy consult if your CAYAs anxiety is elevated.

Music Therapy Tips After Surgery

- Listen to a pre-selected playlist.
- Engage in music making and singing for distraction.
- Ask for a music therapy consult.

Music Therapist

A music therapist uses music therapy to address a person's physical, mental and social needs. They can:

- Help patients manage pain by creating a positive environment with live music at the hospital bedside.
- Engage in distracting musical play and music-guided imagery to help with sleep.

- Provide healthy coping techniques using preferred music for at home after hospital discharge.

Who to Call

The Pediatric Supportive Care Team will help you and your CAYA with an overall plan for pain management. Talk with the Pediatric Supportive Care Team for more information or a referral to any of the services listed in this handout.

Pediatric Supportive Care Team
713-792-6610
Monday through Friday, 8 a.m. to 5 p.m.

After hours and on weekends, call 713-792-5173
