# Supplementary material for: Development of an Enhanced Recovery Program in Pediatric, Adolescent, and Young Adult Surgical Oncology Patients
Source: Children (Basel). 2021 Dec 8;8(12):1154. doi: 10.3390/children8121154 (PMC8700533; doi:10.3390/children8121154)
Supplement: Supplementary file 1 [file children-08-01154-s001.zip › File S2.pdf]

Enhanced Recovery After Surgery
Pediatric Patient

Your child's surgical team has developed a plan to improve their recovery after surgery. Faster healing and return of well-being are goals with enhanced recovery. Following this plan will help reduce problems from surgery, prevent loss of movement and improve pain management. It will also help reduce your child's symptoms and improve their quality of life.

The Day Before Surgery

On the business day (Monday through Friday) **before** surgery, you will receive a call between 5 p.m. and 7 p.m. to tell you what time to check-in for your child's surgery. Be sure staff have your current phone number.

- If you do not receive a call by 7 p.m., call **713-563-8974** before 9 p.m. to get your child's check-in time.
- You must call the Admissions office at **713-792-6136** at least 1 day before your child's surgery to complete the admissions process. The Admissions office is open 24 hours per day, 7 days per week.

Report to the surgery check-in desk at the scheduled check-in time. The surgery check-in desk is located in the **Main Building, Floor 5, near Elevator E**. You will see the check-in desk when you exit the elevator. A staff member will check in your child and give you more information.

If you will be late for any reason on the day of surgery, call **713-792-6420**.

Valet Parking is available near entrance 2 and 3 of the Main Building, 24 hours per day, 7 days per week. If the attendant is not available when you arrive, come into the building and ask the security guard to page the Valet Supervisor.

Preparing for Surgery

To improve your child's health and well-being in the weeks before their surgery, your child should get regular exercise, continue with play activities, maintain a regular sleep schedule and do things to reduce his or her stress and anxiety. Discuss any concerns honestly with your child's care team. Making changes can improve your child's readiness for their surgery.

Consults

The care team will have you and your child meet with a child life specialist, dietitian and physical therapist for further support. If time does not allow for a meeting due to the surgery schedule, you will meet with them after surgery.

The University of Texas MD Anderson Cancer Center ©2021

Restful Sleep
It is important to be well rested before surgery. Your child should get a good night's sleep for several days leading up to the day of surgery. It may be difficult to sleep well in the hospital following surgery because the care team monitors your child closely.

Packing
The following items will help with your child's comfort and symptom control while in the hospital.

Bring these items for your child:
- Toothbrush and toothpaste
- Rubber soled slippers
- Robe
- Undergarments or shorts and relaxed fitting clothes to wear for walking and activity
- Comfort item (blanket, stuffed animal, toy and music)
- Chapstick® or lip moisturizer
- Glasses/contacts/eye mask or cover
- Medical devices (inhalers) and equipment (walking aides/wheelchair)
- Routine medicine in original bottles
- Electronic devices (tablet, iPad)

Leave valuables at home or with a loved one during your child's surgery. If you forget and bring valuables, you may contact hospital security to lock up your belongings. MD Anderson is not responsible for lost valuables.

The Night and Morning Before Surgery

Eating the Night Before Surgery
Eat healthy, well-balanced meals the day before surgery. **No solid foods or candy within 8 hours** of your child's scheduled surgery time, unless instructed otherwise.

Your child may have clear (thin) liquids up to 2 hours before their surgery arrival time. Clear (thin) liquids include water, fruit juices (no pulp), Gatorade®, Sprite®, Pedialyte® and clear broth. No apple cider or drinks with milk or cream.

Also, follow any special nutrition instructions given to you by your child's surgical team.

Preparing Your Child's Skin/Bathing Instructions
Showering well before surgery will help prevent infections after surgery. Your child must shower the night before and the morning of surgery with a special soap called 4% chlorhexidine gluconate (also called CHG or Hibiclens®) or an anti-bacterial soap, such as Dial®. CHG or Hibiclens® can be purchased at most local pharmacy stores.
- **Do not** use these products near your child's face, eyes or ears. Wash their hair and face with normal shampoo/soap and water.
- Wash your child's skin gently and do not scrub too hard. Begin by washing the areas where their surgery will be done.
- Leave the special soap on your child's skin in the area of their surgery for 5 minutes before rinsing.
- After 5 minutes, fully rinse the special soap off their body.

- Pat your child dry with a clean towel. Do not apply lotions, soaps, body washes, deodorants, perfumes, powders, hair products or makeup after your child showers.
- After showering, have your child put on clean clothes.
- On the night before surgery, have your child sleep on clean bed sheets.
- **Do not** use make-up, lotions, perfumes, deodorants or any hair products (hair spray, gel, mousse) on the day of surgery.
- **Do not** shave anywhere on your child's body that is near the surgery area. Shaving this area can increase your child's risk of infection.

The Day of Surgery

Routine Medicines

The anesthesia team will tell you which of your child's routine medicines they may take the morning before surgery. Take medicines with a small sip of water. Your child may brush their teeth and use mouthwash in the morning. Do not chew gum or mints the morning of surgery.

Once your child is in a patient room in the holding area, a nurse will give your child a hospital gown to wear. It is the only thing your child can wear into the operating room. Remove the following items for your child's safety:

- Jewelry, including all body piercings
- Eyeglasses
- Contact lenses
- Prosthesis (eye or leg)
- Bobby pins
- Hair clips
- Wig

Your nurse will direct your child to use the bathroom to empty their bladder. The care team will discuss your child's allergies and any new symptoms or concerns with you. While in the holding area, the care team may give medicine to help your child relax and one or more medicines to help manage nausea and pain after surgery.

Surgery Waiting

One family member may accompany your child to the holding area. When your child goes to the operating room, the care team will direct family to the surgery waiting room.

- There is a limit of 4 visitors per patient in the surgery waiting areas. Space is limited.
- A nurse will come out and give a report on your child's status every 2 to 2 ½ hours starting at 10 a.m. Volunteers are available in the waiting room to answer questions throughout the day.

After Surgery

Your child will wake up in the operating or recovery room. The following surgical items may be connected to your child and will be removed by the care team as soon as it is safe to do so.

- **Urinary catheter –** This is a tube inserted into the bladder to keep it empty.

- **Nasal gastric tube** –This tube goes from the nose down to the stomach to remove gas and fluids from the stomach.
- **Surgical drain** – These tubes remove fluid from the surgical site.
- **Chest tube** – This tube helps drain fluid and air from the chest.

Sitting Up and Getting Out of Bed

After your child is more awake, they will be transferred to a hospital room. On the day of surgery, a care team member, usually a physical and occupational therapist (rehab team), will help your child sit in a chair. If your child's care team decides it is safe for your child to walk, the care team will encourage your child to do so. The rehab team helps your child with movement to and from the bed, walking, pain management and self-care activities, such as dressing, bathing, using the toilet and grooming. This team also provides your child with any aides if needed, such as a cane, walker or wheelchair before hospital discharge.

It is very important that your child participate in these activities to improve their recovery. Your child will be encouraged to sit and be out of the bed for 6 to 8 hours every day, including for meals if able. Your child may receive a referral for outpatient therapy services if more help is needed at discharge.

How You Can Enhance Your Child's Recovery

1. **Report symptoms.** Better symptom control improves your child's recovery. Report symptoms or changes to your child's care team so they can be addressed quickly.

2. **Exercise the lungs.** Your child's nurse will teach your child how to use an incentive spirometer and/or ask your child to do deep breathing exercises. The care team recommends 10 times each hour while awake. This exercise prevents pneumonia and other lung problems after surgery. It is also one of the best things your child can do to help their lungs recover. Have your child sit in the bedside chair in their room. This also helps their lungs recover.

3. **Walk.** Once approved by your child's doctor and physical therapy, walk with your child around the nurse's station several times a day. Encourage your child to walk to the bathroom to do self-care activities every day. It helps your child recover, reduce stiffness and manage pain that is sometimes common after surgery. Walking is one of the best things your child can do to help recover and lower their risk for developing clots in their legs. The care team will also encourage your child to increase activity every day.

4. **Sit up.** Encourage your child to sit up in a chair as much as possible during the day. Sitting also helps exercise your child's lungs, promotes blood circulation, helps with digestion and may also prevent constipation.

5. **Eat and drink as directed.** Your child may slowly advance their diet or be fed a special formula through a feeding tube. If your child eats by mouth, the care team will recommend what foods to avoid and good food options to choose. The medicine your child received during surgery will make them thirsty. Do not let your child over drink or eat. This will not quench

their thirst, but rather leave them feeling bloated or nauseated. Keep your child's lips moist with moisturizer to help reduce dry mouth.

6. **Sleep.** Sleeping is often hard to do in the hospital. Your child's care team will work with you to help not disturb your child's sleep, unless needed. Some patients find it helpful to use earplugs or wear an eye mask. Tell your child's care team if they normally use medicine to help them sleep. Do not give your child any medicines from home. The care team will prescribe the medicines they want your child to take while in the hospital.

7. **Help with blood circulation.** For a child 10 years of age or older, the nurse may fit them with a special compression device to keep on their legs. It massages their calves and promotes healthy blood circulation. It should be worn while your child is in bed. Tell your child's nurse if it is not on or not working properly.

8. **Injections.** Blood clots after surgery are a risk and very harmful. A child older than 10 years of age, may receive a small injection into their belly to prevent blood clots. Your child may need to continue these injections at home after their hospital discharge. Your child's nurse will teach you how to give these injections at home, if needed.

Enhanced Recovery Medicines

Your child will take scheduled medicines (non-opioid) to treat the different types of pain that are common after surgery, such as inflammation and nerve pain. These medicines prevent inflammation and decrease nerve pain that may occur from the surgery. Nerve pain can be described as shooting, traveling or burning pain. If your child's pain is not well controlled, the care team may give pain medicine (opioid pain relievers) as needed to decrease their pain.

Your child's medical team will review their medical history and allergies to make sure these medicines are safe for your child to take. Tell the doctor if your child has allergies.

Pain Management

There are many methods and resources available to help your child, adolescent or young adult manage pain. **Child, Adolescent and Young Adult Life Specialists** are providers with special training who work with caregivers, family and patients. They help provide strategies to support positive coping and ease emotional distress. Developing a pain management plan is one way they can help. This plan may include the following:

- Art and music therapy
- Integrative medicine (aromatherapy, acupuncture and massage therapy)
- Lifestyle strategies (deep breathing, meditation and relaxation, nutrition and sleep hygiene)
- Self-soothing support (Apps, personal resources and positive activities)
- Social work counseling and psychological services
- Spiritual support

Talk with your care team to learn more about these resources and services.

Pain Medicines

The care team will assess your child's pain using a common pain rating scale called the **Wong-Baker Faces® Scale**.

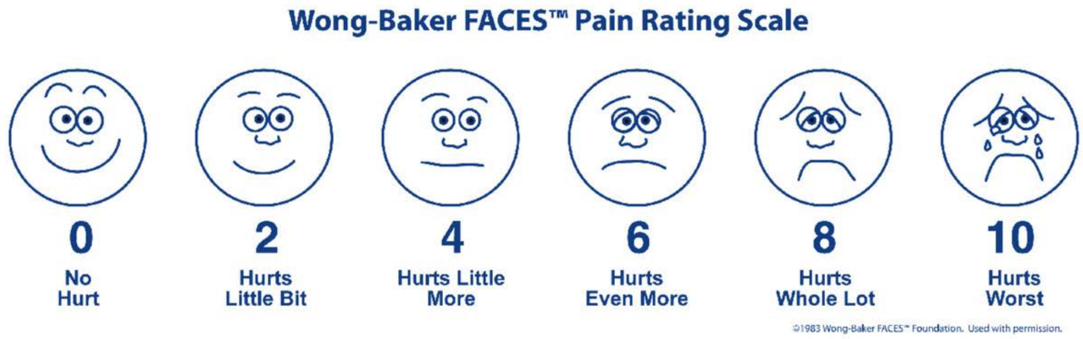

Below are some of the medicines your child's care team may use to help manage their pain.

Non-Opioid Pain Medicine	How it Works	Side Effects and Other Instructions
Acetaminophen (Tylenol®)	This helps to prevent pain and decrease need for opioid medicines when taken on a scheduled basis.	In general, this medicine is well tolerated. Tell your child's care team if any side effects bother your child or if your child has liver disease.<br><br>Avoid other medicines that contain acetaminophen.
Celecoxib (Celebrex®)	This helps to prevent pain and decrease need for opioid medicines when taken on a scheduled basis. This reduces inflammation after surgery. Celecoxib is similar to ibuprofen, but it is gentler on your child's stomach.	Common side effects include nausea and vomiting. Tell your child's team if your child has a history of heart failure, kidney impairment or gastrointestinal bleeding.<br><br>Take with food if it causes an upset stomach.
Ibuprofen (Advil®, Motrin®)	This helps to prevent pain and decrease need for opioid medicines when taken on a scheduled basis. This reduces inflammation after surgery. Ibuprofen is similar to celecoxib.	Common side effects include nausea and vomiting. Tell your child's team if your child has a history of heart failure, kidney impairment or gastrointestinal bleeding.<br><br>Take with food if it causes an upset stomach.

Ketorolac (Toradol®)	This helps to prevent pain and decrease need for opioid medicines when taken on a scheduled basis.	Common side effects include upset stomach, headache and heartburn.
**Opioid Pain Medicine**	**How it Works**	**Side Effects and Other Instructions**
Methadone	This is a long-acting opioid medicine. It can be given by mouth to manage high levels of pain for a longer period of time.	Common side effects include sleepiness, dizziness, itching, confusion, nausea, vomiting and constipation. Tell your child's team if your child has a history of seizures or kidney impairment.
Oxycodone (Roxicodone®)	This is an opioid pain medicine and can be given by mouth when your child's pain level is high and not controlled by their scheduled non-opioid medicines.	Common side effects include sleepiness, dizziness, confusion, nausea, vomiting and constipation.

Resources

Your child will have many care team members caring for them during their hospital stay. To learn more about each person's role, refer to your **Welcome Packet** or the **Hospital Stay Guide** in your child's hospital room.

For a complete list of MD Anderson services, ask your child's care team for a copy of the **Resources and Services** guide.

Enhanced Recovery After Surgery Society
http://www.erassociety.org
