# Supplementary material for: Development of an Enhanced Recovery Program in Pediatric, Adolescent, and Young Adult Surgical Oncology Patients
Source: Children (Basel). 2021 Dec 8;8(12):1154. doi: 10.3390/children8121154 (PMC8700533; doi:10.3390/children8121154)
Supplement: Supplementary file 1 [file children-08-01154-s001.zip › File S3.pdf]

Enhanced Recovery Program Overview for Pediatric Patients

Prepare for Surgery	Day Before Surgery	Day of Surgery	After Surgery
**General Instructions:**<br>• Share details of medical conditions, implanted devices, and surgical and medical history with your child's care team.<br>• Discuss emotional concerns or social issues that might slow recovery.<br>• Meet with a child life specialist for support.<br>• Follow nutrition instructions.<br>• Practice deep breathing (inhale and exhale slowly, 10 times) every hour while awake. Review use of an incentive spirometer.<br>• Follow bowel prep, skin prep and special bathing instructions, if directed.<br>• Choose or arrange a caregiver for recovery support.	**General Instructions**:<br>• Eat a well-balanced lunch and dinner. No solid foods or candy within 8 hours of scheduled surgery time, unless instructed otherwise.<br>• Get restful sleep.<br>• Pack items that will help with your child's comfort while in the hospital.<br>• Leave valuables at home.<br>• Shower the night before and sleep on clean sheets.<br>• Take prescribed pre-surgery medicines.	**Diet**: If instructed, drink apple, grape or cranberry juice, chicken or beef broth or carb loading drink 2 hours before arrival for surgery. **No milk or juices with pulp**. **Follow printed or care team instructions.**<br><br>**Medicines:** Bring home medicines and any medical devices (inhalers) for review and use if needed before or just after surgery. You may keep them or the care team will lock them up and return to you at discharge.<br><br>**Surgery holding area**: Your child may receive medicine to help them relax and one or more medicines to help manage nausea and pain after surgery.<br><br>**Family and friends**: May stay in the waiting area while your child is in the operating room.	**Pain Management:** Main goal is to provide your child with adequate pain management using medicine and other methods. This allows your child to walk and do deep breathing exercises with minimal side effects.<br><br>**Mobility:** Your child is encouraged to safely walk, move around, play and do self-care activities as able. Expect your child to be out of bed many times per day and sit for 6 to 8 hours every day. This helps with bowel function, improves circulation, prevents lung infections and promotes healing.<br><br>**Urinary catheter**: If your child has a urinary catheter, it will be removed as soon as possible.<br><br>**Diet**: Start with clear liquids and advance as instructed on day of surgery. Avoid greasy foods.<br><br>**Exercise lungs:** Use incentive spirometer 10 times every hour while awake, until discharge.

Prepare for Surgery	Day Before Surgery	Day of Surgery	After Surgery
**Home Medicines**: Follow instructions given by the Perioperative Evaluation Management Center (POEM) for medicines to take or not take the night before and morning of your child's surgery.<br><br>**Physical Medicine**: Begin a gentle fitness routine if not already doing so. Play and walking in or around the home is also considered exercise. If cleared by the doctor and age appropriate, your child may lift light weights to improve their strength. Your child's doctor may suggest meeting with a physical or occupational therapist before surgery.		**Before your child goes into operating room**:<br>• Remove their clothing, contact lenses, jewelry, etc. and put on gown.<br>• Nurse will direct your child to the bathroom.<br>• Care team will review medicines, devices, allergies, symptoms and concerns.<br><br>**During surgery:** Nurse will come to waiting area to give family updates.<br><br>**What to expect right after surgery:**<br>• Surgeon will talk with family members.<br>• Your child will wake-up in the recovery room.<br>• The care team will help your child get out of bed after surgery. Movement helps the healing process.<br>• Your child may have a urinary catheter to empty bladder.<br>**Transfer:** Your child will be moved to a hospital room when awake and stable.	**Blood circulation**: For a child 10 years of age or older, a special compression device may be used to massage calves. Movement also helps with circulation.<br><br>**Discharge Criteria:**<br>• Your child resumes regular diet as able to tolerate or follow instructions for other nutrition options if directed.<br>• Pain is controlled with oral medicines.<br>• Able to urinate and show signs of bowel function.<br>• Able to do self-care activities.<br>• Able to move and walk safely around the nurse's station at least 2 times.<br>• Discharge planning, education and follow-up care instructions.<br><br>Goal for discharge time is 11:00 a.m.

The University of Texas MD Anderson Cancer Center ©2021
