# Supplementary material for: Development of an Enhanced Recovery Program in Pediatric, Adolescent, and Young Adult Surgical Oncology Patients
Source: Children (Basel). 2021 Dec 8;8(12):1154. doi: 10.3390/children8121154 (PMC8700533; doi:10.3390/children8121154)
Supplement: Supplementary file 1 [file children-08-01154-s001.zip › File S4.pdf]

Nutrition

Pediatric Enhanced Recovery After Surgery

Nutrition is very important for your child during this time. A diet that provides good nutrition before and after surgery can help your child recover faster and help prevent infections and problems with wound healing. Follow these guidelines if your child's care team clears your child for a regular diet.

The Day Before Surgery

Be sure your child eats a well-balanced meal for dinner that includes protein rich food, such as lean meats, fish, poultry, eggs, beans, nuts or reduced or low-fat dairy products. It is also important that your child drink plenty of fluids and be well hydrated before surgery.

Carbohydrate Drink

Your child may need to drink a carbohydrate drink the night before surgery and the day of surgery. Your child's care team will review this information with you and let you know which directions to follow.

☐ Do not follow carb loading instructions for your child's surgery.

☐ Follow carb loading instructions for your child's surgery.

Your child weighs ________ and needs to drink ________mL of carbohydrate drink.

Your child's care team will calculate the amount your child needs to drink and write it down on the charts below. (Amount is 3 to 5 mL/kg of your child's weight.)

Choose 1 of the options below and drink in the **evening or before bedtime.**
(**Do not** choose diet or light options if you drink the apple, cranberry, or grape juice.)

Chart 1. Night before surgery

Carbohydrate Drink	Amount	When to Drink
Gatorade or Powerade	Follow directions based on your child's weight	
Pedialyte		
Clearfast Pre-op or Ensure Pre-surgery	____________mL	Evening or before bedtime
Apple or cranberry juice		
Grape juice		

The University of Texas MD Anderson Cancer Center ©2021

The Day of Surgery

No solid foods or candy within 8 hours of your child's scheduled surgery time, unless instructed otherwise. Your child may have clear (thin) liquids up to 2 hours before their surgery. Clear (thin) liquids are liquids you can see through when poured into a glass, such as water, tea, sports drinks, and juice with **no pulp**. **No drinks with cream or milk**.

Stop all liquids 2 hours before surgery.

Carbohydrate Drink

If instructed by your child's care team, follow the carb loading instructions.

Choose 1 of the options below and drink **2 hours before** surgery**.**
(**Do not** choose diet or light options if you drink the apple, cranberry, or grape juice.)

Make sure your child drinks just before coming to the hospital. However, keep in mind the distance you will travel to the hospital so that your child follows the **2 hours before** you arrive for surgery instructions.

Chart 2. Day of surgery

Carbohydrate Drink	Amount	When to Drink
Gatorade or Powerade	Follow directions based on your child's weight<br><br>___________mL	Up to 2 hours before surgery
Pedialyte		
Clearfast Pre-op or Ensure Pre-surgery		
Apple or cranberry juice		
Grape juice		

After Surgery

After surgery, your child will need more nutrients to help their body heal. Depending on your child's type of surgery, he or she may be on a special diet. Talk with your care team about any special diet guidelines or restrictions your child needs to follow. Otherwise, make sure your child eats smaller meals more often that include lean proteins, such as yogurt, cottage cheese, milk, chicken without skin, fish and healthy fats, such as avocado, olive and nut butters.

Nutrition Supplements

You may need to start your child on an oral nutrition supplement if your child is having a hard time with eating or weight loss. Ask to speak with the dietitian to discuss whether this is a good choice for your child.

For questions, talk with your care team or you may call 713-792-2254 to speak with a dietitian.

The University of Texas MD Anderson Cancer Center ©2021
