# Supplementary material for: Development of an Enhanced Recovery Program in Pediatric, Adolescent, and Young Adult Surgical Oncology Patients
Source: Children (Basel). 2021 Dec 8;8(12):1154. doi: 10.3390/children8121154 (PMC8700533; doi:10.3390/children8121154)
Supplement: Supplementary file 1 [file children-08-01154-s001.zip › File S5.pdf]

Prehabilitation
Pediatric Enhanced Recovery After Surgery

Taking part in an exercise program before surgery can help with your child's recovery after surgery. This is called **prehabilitation** or **prehab**. The program is tailored to your child's care needs, with the goal of getting your child in the best possible physical and mental shape for surgery and helping with their recovery. The prehab team may include physicians, surgeons, physical therapists, occupational therapists, dietitians, and other medical providers.

Benefits

Patients who participate in prehab before surgery improve their chances of having a faster recovery. It may also reduce the chances of problems after surgery.

Studies show that patients who do prehab:
- Have a better quality of life during pre-surgery treatments (chemotherapy or radiation) and after surgery
- Regain strength and physical function faster and more easily after surgery
- Are able to return to daily activities faster and more easily after surgery
- Stay in the hospital for a shorter period of time
- Are less likely to suffer from social isolation and perform better in school
- Have more coping strategies for managing pain and cancer-related fatigue

Your child will have a prehab team, and this team works closely with your child's surgical team before their scheduled surgery. Your child will continue to do prehab until surgery. It is important that your child has time to improve their physical condition and prepare their body to handle the stress of surgery.

Appointments

Your child will go to Rehabilitation Services for their appointments. It is located in the Main Building, Floor 1, near Elevator F, G1.3418. Be sure to follow your child's prehab plan and go to all of their appointments.

Resources

Watch this video to learn about exercises for kids, teenagers and young adults with a cancer diagnosis. **https://www.youtube.com/watch?v=cc435ONdnFY&t=316s**

Enhanced Recovery After Surgery Society
http://www.erassociety.org/

The University of Texas MD Anderson Cancer Center ©2021
